# Perceptual Discrimination of Basic Object Features Is Not Facilitated When Priming Stimuli Are Prevented From Reaching Awareness by Means of Visual Masking

**DOI:** 10.3389/fnint.2018.00013

**Published:** 2018-04-19

**Authors:** Hayden J. Peel, Irene Sperandio, Robin Laycock, Philippe A. Chouinard

**Affiliations:** ^1^Department of Psychology and Counselling, School of Psychology and Public Health, La Trobe University, Melbourne, VIC, Australia; ^2^School of Psychology, University of East Anglia, Norwich, United Kingdom; ^3^School of Health and Biomedical Sciences, RMIT University, Melbourne, VIC, Australia

**Keywords:** vision, form discrimination, orientation discrimination, size discrimination, visual masking, priming

## Abstract

Our understanding of how form, orientation and size are processed within and outside of awareness is limited and requires further investigation. Therefore, we investigated whether or not the visual discrimination of basic object features can be influenced by subliminal processing of stimuli presented beforehand. Visual masking was used to render stimuli perceptually invisible. Three experiments examined if visible and invisible primes could facilitate the subsequent feature discrimination of visible targets. The experiments differed in the kind of perceptual discrimination that participants had to make. Namely, participants were asked to discriminate visual stimuli on the basis of their form, orientation, or size. In all three experiments, we demonstrated reliable priming effects when the primes were visible but not when the primes were made invisible. Our findings underscore the importance of conscious awareness in facilitating the perceptual discrimination of basic object features.

## Introduction

Many studies have demonstrated that the processing of visually presented faces (Kouider et al., 2009; Faivre et al., 2012), words (De Houwer et al., 2002; Klauer et al., 2007; Ortells et al., 2016) and numbers (Dehaene et al., 1998; Naccache and Dehaene, 2001) can occur outside of awareness. Less research has considered how more basic features of objects might be processed outside of awareness. Thus, we aimed to determine if different types of basic stimulus features can be processed outside of awareness. More specifically, we used a priming paradigm to determine if the subconscious processing of stimulus form, orientation and size could alter the subsequent conscious perception of another stimulus.

The ventral pathway is essential for the perceptual recognition of objects (Goodale and Milner, 1992, 2013). Early visual areas are known to be important in processing basic low-level stimulus features while later ones are more concerned with more complex features (Tanaka, 1993). Electrophysiological studies in nonhuman primates and functional magnetic resonance imaging (fMRI) studies in humans have demonstrated that the primary visual cortex (V1) can process information about the size and orientation of stimuli (for a review see Grill-Spector et al., 2001). As information is processed further along the ventral stream, neurons have larger receptive fields and show preferential activation for more complex stimuli (Brincat and Connor, 2004; Yau et al., 2013). How these visual areas interact and how these interactions are implicated in the conscious perception of stimuli is not fully understood. Some propose that conscious perception occurs mostly in a bottom-up, feedforward manner (Marr, 1982; Riesenhuber and Poggio, 2000) while others highlight the importance of top-down modulation (Rao and Ballard, 1999; Bar, 2003). Regardless of the nature of these interactions, the question remains as to whether or not initial forms of processing carried out before conscious awareness can ultimately influence perceptual recognition.

Contemporary models of consciousness attempt to explain the differences in neural activation when stimuli are perceptually visible compared to when they are not—allowing conscious and subconscious processes to be disentangled with increasing precision. For instance, *Recurrence Theory* (Lamme and Roelfsema, 2000) proposes that perceptually invisible stimuli reflect a feedforward response that fails to trigger the necessary recurrent processing needed for stimuli to become visible. In contrast, the *Global Neuronal Workspace Theory* (Dehaene and Changeux, 2011) proposes that perceptually invisible stimuli reflect a signal that is too weak to be globally broadcasted elsewhere in the brain. Within the latter framework, Dehaene et al. (2006) and Dehaene and Changeux (2011) differentiated between *subliminal* and *preconscious* processing. Namely, subliminal processing, where the bottom-up processing of a stimulus is insufficient to reach the threshold for conscious perception, and preconscious processing, where a stimulus is potentially visible but is not perceived due to distraction or inattention (Dehaene and Changeux, 2011). In either case, both have the potential to influence higher level operations (Koch and Tsuchiya, 2006).

Behavioral research has sought to examine these subconscious traces using visual masking (e.g., Breitmeyer, 2015). This method consists of presenting a stimulus to participants rapidly before and/or after a mask consisting of visual noise (Enns and Di Lollo, 2000). The masks are thought to interfere with the bottom-up strength of an incoming stimulus, which prevents further analysis of this signal from reaching conscious awareness. For this reason, masking is used as an experimental method to present stimuli subconsciously in a subliminal state. Visual masking is sometimes combined with a priming paradigm to infer the degree of processing depth of the masked stimulus (Kouider and Dehaene, 2007; Yang et al., 2014). Traditional priming paradigms (without visual masking) consist of presenting a target stimulus preceded by the presentation of an earlier stimulus called the prime. The target is processed faster when the prime and target share some common perceptual or semantic feature (Tulving and Schacter, 1990). Following this logic, one can infer that a masked prime was processed outside of awareness if it exerts an influence on the target. Indeed, presenting a masked prime that is either perceptually or semantically congruent with a target is frequently used as a litmus test for determining subtle subconscious influences in perceptual and semantic decision-making (e.g., Ortells et al., 2016).

It is quite evident from a number of studies that invisible primes can facilitate the perceptual recognition and classification of visible holistic images into different semantic categories (e.g., Dell’Acqua and Grainger, 1999; Almeida et al., 2008; Eddy and Holcomb, 2009; Van den Bussche et al., 2009; Sakuraba et al., 2012; Hesselmann et al., 2016). However, the question remains as to whether or not information about the basic form, orientation and size of objects can also be processed outside of awareness for the purposes of facilitating perceptual recognition for those features as opposed to their holistic properties. There is preliminary evidence that it can.

In a recent study, Jimenez et al. (2017) had participants classify visible targets as being either a square or a diamond following the presentation of perceptually invisible primes that consisted of either the same or the alternative shape. The authors found that subliminal priming effects were indeed observed. Likewise, Hesselmann et al. (2016) demonstrated that a prime’s shape as opposed to its semantic category *per se* was the principal driver facilitating the classification of animal and tool stimuli as being either elongated or non-elongated. In terms of orientation, several studies demonstrate that the orientation of invisible primes can affect the subsequent perceptual discrimination of a visible target’s orientation (Soto et al., 2011; Montoro et al., 2014; Peremen and Lamy, 2014; King et al., 2016). In terms of size, our earlier work demonstrates opposite findings (Laycock et al., 2017). Namely, the size of invisible primes had no effect on the subsequent perceptual discrimination of a visible target’s size—at least when continuous flash suppression (CFS) was used to render the primes perceptually invisible. It is unknown whether or not visual masking would yield similar results.

As far as we know, the systematic examination of shape, orientation and size has not been examined and compared in one study. Therefore, we aimed to determine if the subconscious processing of basic stimulus features, such as form, orientation and size, could still change the subsequent conscious perception of another stimulus. It was hypothesized that priming would be observed in both the perceptually visible and invisible viewing conditions for each of the three features. If these predictions hold then this would suggest that the perceptual discrimination of these features does not necessitate awareness nor the recurrent feedback mechanisms implicated in conscious awareness discussed earlier (Lamme and Roelfsema, 2000; Dehaene et al., 2006; Dehaene and Changeux, 2011).

## Materials and Methods

### Overview

Participants completed a single testing session that took approximately 1 h and 30 min to complete. The session began with tests of handedness (Oldfield, 1971) and visual acuity (Snellen, 1862). Participants then completed the *form*, *orientation* and *size* experiments in a counterbalanced order as determined by a Latin square. Each experiment consisted of four separate tasks. Participants first completed the threshold task, which established the maximum luminance contrast at which stimuli could be suppressed reliably under visual masking. This was followed by the first recognition task, which was used to verify the lack of perceptual visibility of the masked stimuli. Then, participants completed the priming task, which tested whether or not there was any processing advantage (i.e., improved reaction time) when responding to feature-congruent as opposed to feature-incongruent prime-target combinations, during both visible and perceptually invisible conditions. Each experiment ended with the second recognition task, which was implemented as a way to verify that there were no changes in the visibility of the masked stimuli.

### Participants

We tested 38 participants (16 males, *M*_age_ = 22.39 years, age range: 18–31). Both the size and shape experiments had 34 participants. In the orientation experiment, suppression could not be achieved in four participants. Therefore, these individuals were replaced by four participants so that all three experiments would be matched in sample size. Participants had to be in good health, right-handed and have normal or corrected-to-normal vision. Preference using the right hand was confirmed using a modified version of the Edinburgh Handedness Inventory (Oldfield, 1971) and visual acuity was confirmed using the Snellen chart (Snellen, 1862). Acuity was 20/40 or better in each eye for all participants. Participants provided informed written consent prior to participation and were compensated with a gift card for their time and any inconveniences. This study was approved by the La Trobe University Human Ethics Committee in accordance with the Declaration of Helsinki.

### Stimuli and Apparatus

**Figure 1:**
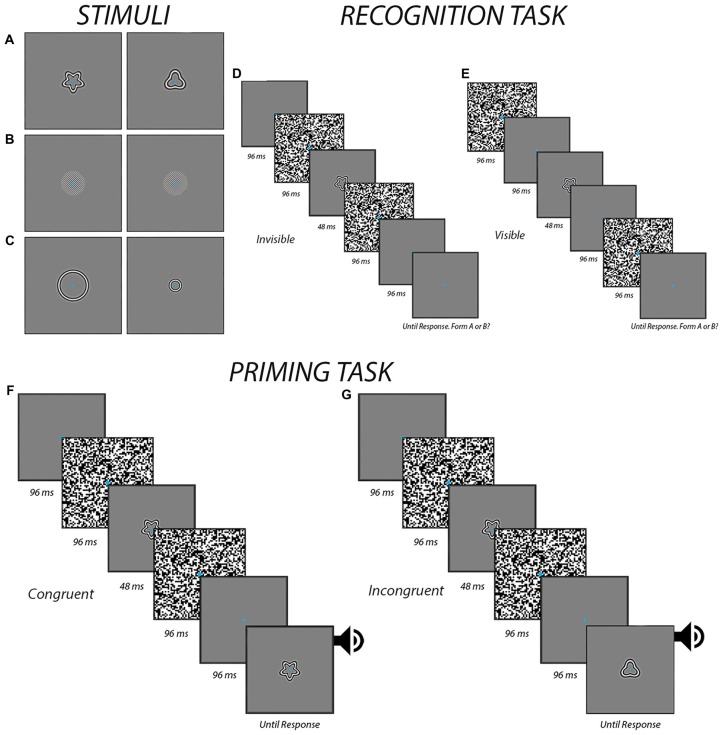
The procedures. The panels display the different form **(A)**, orientation **(B)** and size **(C)** stimuli that were used in the different experiments. Also shown are the temporal sequence of events for both the recognition task during the invisible **(D)** and visible **(E)** conditions and the priming task during congruent **(F)** and incongruent **(G)** trials when the prime was presented subliminally. In the visible condition for the priming task, the masks were presented differently, in a manner similar to the visible condition in the recognition task **(E)**. An auditory alerting cue was presented in the priming task at the end of the trial to help ensure that participants responded to the target image as opposed to the prime.

As shown in Figures 1A–C, the stimuli consisted of two exemplar images for each experiment. They were presented on a gray background of 40.3 c/m^2^, which corresponded to the mean luminance of the entire display. For the form experiment, radial frequency patterns subtending a visual angle of 6.5° were used (Wilkinson et al., 1998; Figure 1A). They were created using in-house programs in Matlab (Mathworks, Natick, MA, USA). The first stimulus had a radial modulation amplitude of 0.25, a radial frequency of 5, and an angular phase of 0° while the second stimulus had a radial modulation amplitude of 0.25, a radial frequency of 3, and an angular phase of 0°. For the orientation experiment, circular Gabor patches were created using the “online Gabor-patch generator” (Mathôt, 2017) and subtended a visual angle of 6.5°, with a spatial frequency of 2.8 cycles per degree. The first Gabor patch was oriented 315° while the second one was oriented 45° (Figure 1B). For the size experiment, the stimuli were created using the same in-house programs as those to generate the stimuli for the form experiment. Stimuli in the size experiment were circles of two different sizes with a radial frequency of 0. The larger circle subtended a visual angle of 9° while the smaller circle subtended a visual angle of 4° (Figure 1C). The stimuli were presented on a 24-inch LCD Dell monitor at a resolution of 1920 × 1200 and a viewing distance of 57 cm. Participants used a chin rest to ensure consistent head positioning. A Dell desktop PC running Windows 7 ran the experiments using E-prime 2.0 software (Psychology Software Tools, Sharpsburg, PA, USA) to deliver the stimuli and record button responses via a model 200a Serial Response Box (Psychology Software Tools, Sharpsburg, PA, USA).

### Threshold Task

The threshold task began by introducing the participants with two stimuli and telling them that they were required to indicate at every trial which of the two are presented. After these instructions were given, a single descending 1-up 1-down staircase procedure was used to determine the maximum luminance contrast at which each stimulus category could be reliably suppressed from awareness using our masking procedure (for details regarding the masking, see procedures for the recognition task in the next section below). For each trial, participants were presented with one of the two stimuli chosen at random by E-prime. They had to verbally say which one they thought they saw and guess if they were unsure. The experimenter then manually entered their response by keypress. The target stimulus was first set at 100% Michelson (1995) luminance contrast. Following each correct categorization, the contrast decreased by 5%. Following an incorrect response, a reversal would occur, meaning the luminance contrast would increase by 5%. The task was programmed to terminate following 12 reversals and the final threshold was calculated as the average of the last eight switches to account for greater potential errors due to uncertainty for the first four.

### Recognition Tasks

The recognition tasks were used to determine if participants were able to perceive the stimuli at the contrast level determined in the previous task. The luminance contrast for all stimuli was set 5% below the threshold derived (i.e., 20% dropped to 15%) in the threshold task to a minimum of 5%. The task began by introducing participants the two stimuli and telling them that they were required to indicate at every trial which of the two are presented. Participants viewed a total of 64 individual trials, which consisted of 32 invisible and 32 visible trials that were presented in a random order. A break was provided midway. The presentation sequences are depicted in Figures 1D,E. Each trial began with the participant maintaining fixation on a blank screen for 3000 ms. This was then followed by the presentation of the stimulus between two visual masks. The timing of the visual masks varied between the visible and invisible trails. At the end of each trial, participants indicated by button pressing which of the two possible stimuli they saw. Participants were told to give their best guess in the event that they did not see anything—as would be expected in the invisible condition. Before moving to the priming task, a preliminary analysis was conducted to verify that accuracy was within the confines of chance (i.e., between 11 and 20 for 32 trials based on a binomial distribution). Chance-level accuracy was taken as evidence that the stimuli were appropriately suppressed and imperceptible (Kingdom and Prins, 2016). If accuracy was outside the limits of chance, luminance contrast was further adjusted (i.e., decreased by an additional 5% if above chance, or increased by 5% if below chance) and the recognition task was performed again before moving on to the priming task.

### Procedures for the Priming Task

This priming task was used to determine whether or not feature-congruency had any influence on reaction times to a target following a prime that was either visible or perceptually invisible by means of masking. In total, there were 80 trials: 20 visible congruent trials, 20 visible incongruent trials, 20 invisible congruent trials and 20 invisible incongruent trials. A break was provided midway. The presentation sequences are depicted in Figures 1F,G. The sequence of events was similar to those in the recognition task except with the additional presentation of a visible target at the end of each trial. The participant indicated with button pressing which of two possible images the target corresponded to as quickly and accurately as possible. The target was accompanied by a 100 ms auditory alert cue to help ensure participants were responding to this stimulus as opposed to the prime.

### Statistical Analyses

The data were analyzed using the Statistical Package for Social Sciences (SPSS) version 23 (IBM Corporation; Armonk, NY, USA), JASP software version 0.8 (University of Amsterdam, Amsterdam, Netherlands), and GraphPad Prism version 6 (GraphPad Software Inc., La Jolla, CA, USA). Threshold values were recorded during the procedures.

One-sample *t* tests against a test value of 16 (denoting chance) determined if responses differed significantly from this value for both visible and invisible conditions in the recognition tasks before and after the priming task. For the priming task in each of the three different experiments, the mean reaction time, calculated from only the correct trials, acted as the dependent variable in a two-way repeated-measures analysis of variance (ANOVA) with Visibility (Invisible vs. Visible) and Congruency (Congruent vs. Incongruent) as factors. Effect sizes (partial eta-squared; $\eta_{\text{p}}^{2}$) obtained from the ANOVA are reported. Tukey’s honest significant difference (HSD) pair-wise comparison tests, which corrected for multiple comparisons, were carried out to further examine interactions and effects deemed significant by the ANOVA. In addition, Cohen’s *d* effect sizes for pair-wise comparisons were calculated as the difference between the two means divided by their pooled standard deviation (Cohen, 1988). Unless specified otherwise, all reported *p* values were corrected for multiple comparisons based on an alpha level of *α* = 0.05.

In addition to null hypothesis statistical testing (NHST), Bayes factors were calculated. Within the framework of Bayesian statistics, one quantifies the evidence in support for either the null or the alternative hypothesis relative to the other (Wetzels et al., 2011). This allows the possibility to draw inferences about the viability of the null hypothesis, which traditional NHST cannot do. For this study, we calculated Bayes factors (BF_01_) denoting the likelihood of the null over the alternative hypothesis for the contrasts between congruent and incongruent trials for both the visible and invisible viewing conditions. A BF_01_ value of 3 or more was considered to provide substantial support for the null hypothesis (i.e., an absence of priming) and values less than 0.33 to provide substantial support for the alternative hypothesis (i.e., a presence of priming; Jarosz and Wiley, 2014).

## Results

### Thresholds for the Form, Orientation and Size Experiments

For form, thresholds ranged between 5% and 40% luminance contrast with a mean value of 20.12 (*SD* = 8.26). This was qualitatively higher than for the other two categories with orientation and size having lower values of *M* = 7.05 (*SD* = 5.98, range = 1.25–31.14) and *M* = 7.50 (*SD* = 4.80, range = 1.25–18), respectively.

## Form Experiment

The masking technique used was successful in rendering stimuli perceptually invisible—with categorization accuracy being at chance-level when responding to the masked prime images in both recognition tasks. However, a priming effect was observed only when the primes were visible but not when the primes were masked.

### Recognition Tasks

**Figure 2:**
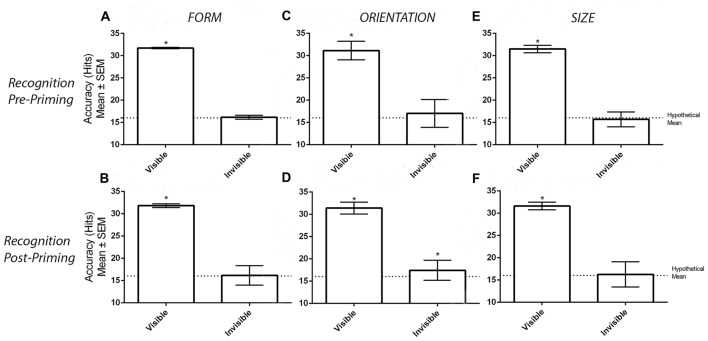
Results from the recognition tasks. Panels **(A,B)** display the mean ± SEM hits in the pre- and post-priming recognition tasks in the form experiment. Similarly, panels **(C,D)** display the hits in the orientation experiment while panels **(E,F)** display the hits in the size experiment. Asterisks (*) denote significant effects at *p* < 0.05.

Figure 2A displays the number of correct hits when participants categorized visible and invisible stimuli before the priming task took place. One-sample *t*-tests demonstrated that the number of correct hits was greater than chance in the visible (*M* = 31.68, *SD* = 0.81, *t*_(33)_ = 113.40, *p* < 0.001) but not invisible (*M* = 16.15, *SD* = 2.65, *t*_(33)_ = 0.32, *p* = 0.749) conditions. Likewise, the post-priming recognition task generated similar results (Figure 2B). Again, one-sample *t*-tests demonstrated a greater number of correct hits compared to chance in the visible (*M* = 31.79, *SD* = 0.41, *t*_(33)_ = 224.40, *p* < 0.001) but not in the invisible (*M* = 16.15, *SD* = 2.18, *t*_(33)_ = 0.39, *p* = 0.696) conditions.

### Priming Task

**Figure 3:**
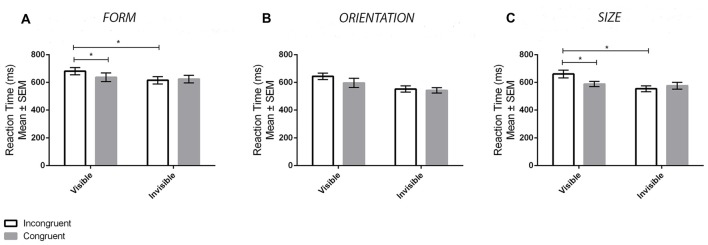
Results from the priming tasks. The panels display the mean ± SEM reaction times in the priming task for both congruent (gray bars) and incongruent (white bars) trials in the visible and invisible conditions for the Form **(A)**, Orientation **(B)** and Size **(C)** experiments. Asterisks (*) denote significant effects at *p* < 0.05.

Figure 3A displays the mean reaction times for the different conditions for the form experiment. ANOVA revealed a main effect of Visibility (*F*_(1,33)_ = 16.68, *p* < 0.001, $\eta_{\text{p}}^{2}$ = 0.34), denoting slower reaction times in the visible compared to invisible conditions, but not a main effect of Congruency (*F*_(1,33)_ = 1.82, *p* = 0.187, $\eta_{\text{p}}^{2}$ = 0.05). In addition, there was a significant Visibility × Congruency interaction (*F*_(1,33)_ = 9.48, *p* = 0.004, $\eta_{\text{p}}^{2}$ = 0.22). Tukey’s HSD tests revealed that this interaction was driven by a priming effect in the visible (*p* = 0.006, *d* = 0.26) but not invisible (*p* = 0.891, *d* = 0.06) conditions. Specifically, there were differences between congurent and incongruent trials for the former but not the latter. Bayes factors were also calculated for contrasts between congruent and incongruent reaction times. A value of BF_01_ = 4.19 was found for the invisible contrasts, providing substantial support for no priming effects. In contrast, a value of BF_01_ = 0.52 was found for the visible contrasts, which does not provide substantial support for the alternative hypothesis. Accuracy results at the group level were at ceiling levels of performance (*M* = 97.87%, *SD* = 3.48%, range: 85% to 100%). Thus, accuracy was not compared between conditions. Nonetheless, the presence of a possible speed-accuracy trade-off effect was still assessed by averaging both accuracy and reaction time scores across conditions for each participant and then correlating them (Wickelgren, 1977). No significant correlation was found (*r*_(32)_ = 0.06, *p* = 0.749), demonstrating a lack of a speed-accuracy trade-off. In summary, priming ocurred in the visible but not invisible conditions.

## Orientation Experiment

The priming task in this experiment also demonstrated priming in the visible but not invisible conditions. Although the recognition task performed prior to the priming task revealed that the stimuli were effectively suppressed from conscious awareness, the same procedure performed after the priming task suggests that the stimuli were no longer suppressed from awareness as effectively.

### Recognition Tasks

Figure 2C displays accuracy for the visible and invisible conditions. Before the priming task, the number of correct hits differed from chance in the visible (*M* = 31.12, *SD* = 2.07 *t*_(33)_ = 42.57, *p* < 0.001) but not invisible (*M* = 17.00, *SD* = 3.14, *t*_(33)_ = 1.89, *p* = 0.073) conditions. However, this was no longer the case when the same recognition procedure was carried out after the priming task. As demonstrated in Figure 2D, one-sample *t* tests revealed that the number of correct hits were greater than chance in both visibility conditions (Visible: *M* = 31.38, *SD* = 1.34 *t*_(31)_ = 65, *p* < 0.001; Invisible: *M* = 17.72, *SD* = 2.24, *t*_(31)_ = 3.59, *p* = 0.001). Two participants were unable to provide recognition measures due to time constraints. Therefore, data from only 32 participants were considered.

### Priming Task

Figure 3B displays the mean reaction times for the different conditions in the orientation experiment. ANOVA revealed a main effect of Visibility, such that reaction times in the visible condition were slower than the invisible condition (*F*_(1,33)_ = 47.23, *p* < 0.001, $\eta_{\text{p}}^{2}$ = 0.58), as well as a main effect of Congruency, such that reaction times in the incongruent condition were slower than those in the congruent condition (*F*_(1,33)_ = 16.44, *p* < 0.001, $\eta_{\text{p}}^{2}$ = 0.35). There was no interaction between Visibility and Congruency (*F*_(1,33)_ = 2.35, *p* = 0.135, $\eta_{\text{p}}^{2}$ = 0.08). The lack of an interaction is most likely a power issue as opposed to the presence of a priming effect in both the visible and invisible conditions. Performing paired sample *t*-tests comparing congruent and incongruent conditions reveal significant priming in the visible (*t*_(33)_ = 2.4, *p* = 0.022) but not invisible (*t*_(33)_ = −1.1, *p* = 0.278) condition. Furthermore, analysis of the data using a Bayesian approach corroborates this interpretation. Specifically, there was substantial support for priming in the visible condition (BF_01_ = 0.21) and substantial support for no priming in the invisible condition (BF_01_ = 3.45). Accuracy results were again at ceiling levels of performance (*M* = 97.76%, *SD* = 4.97%, range: 60% to 100%). Therefore, this dependent variable was not analyzed except for determining whether or not there was a speed-accuracy trade-off effect. No significant correlation was found between reaction times and accuracy (*r*_(32)_ = −0.02, *p* = 0.900).

## Size Experiment

The size stimuli were effectively masked. Recognition procedures performed before and after the priming task demonstrated no differences in the number of correct hits from chance in the invisible viewing conditions. Similar to the priming tasks in the form and orientation experiments, priming effects were observed in the visible but not invisible conditions.

### Recognition Tasks

Figure 2E displays the number of correct hits in the visible and invisible conditions before the priming task. One-sample *t*-tests revealed that the number of corrects hits were greater than chance in the visible (*M* = 31.47, *SD* = 0.83, *t*_(33)_ = 109.30, *p* < 0.001) but not invisible (*M* = 15.68, *SD* = 1.67, *t*_(33)_ = 1.13, *p* = 0.265) conditions. The subsequent suppression check conducted after the priming task confirmed that the perceptual invisibility of the size stimuli was maintained. As displayed in Figure 2F, one-sample *t*-tests demonstrated a greater number of correct hits compared to chance in the visible (*M* = 31.59, *SD* = 0.86, *t*_(33)_ = 106.10, *p* < 0.001) but not invisible (*M* = 16.24, *SD* = 2.83, *t*_(33)_ = 0.49, *p* = 0.631) conditions.

### Priming Task

Figure 3C displays the mean reaction times for the different conditions in the size experiment. ANOVA revealed a main effect of Visibility, whereby reaction times for the visible condition were slower in comparison to the invisible one (*F*_(1,33)_ = 34.80, *p* < 0.001, $\eta_{\text{p}}^{2}$ = 0.51). There was also a main effect of Congruency, whereby reaction times were faster in the congruent compared to the incongruent conditions (*F*_(1,33)_ = 5.08, *p* = 0.031, $\eta_{\text{p}}^{2}$ = 0.13). An interaction between Visibility and Congruency was also found (*F*_(1,33)_ = 16.62, *p* < 0.001, $\eta_{\text{p}}^{2}$ = 0.34). Tukey HSD tests demonstrated that this interaction effect was driven by priming effects in the visible (*p* = 0.002, *d* = 0.53) but not invisible (*p* = 0.553, *d* = 0.16) conditions. Using a Bayesian approach, a value of BF_01_ = 1.64 was found for the invisible contrast, and a value of BF_01_ = 0.02 was found for the visible contrast. This provides substantial evidence in favor of priming for the visible condition; however, the analysis is inconclusive regarding the presence or absence of priming in the invisible condition. Accuracy results were at ceiling levels of performance again (*M* = 98.05%, *SD* = 3.66%, range: 80% to 100%) and therefore were not analyzed except for the purposes of assessing the presence of a speed-accuracy trade-off effect. No significant correlation was found (*r*_(32)_ = −0.34, *p* = 0.052) indicating the absence of a speed-accuracy trade-off.

## Discussion

We hypothesized that priming effects would persist when the primes were rendered perceptually invisible by means of visual masking. This hypothesis was not supported. Visual processing of subliminally presented primes did not influence one’s ability to classify targets based on their shape, orientation and size—even though priming effects were observed in the visible condition. The presence of priming effects during the visible condition demonstrates that the method of presentation was effective for determining the presence of priming effects. Thus, the lack of effects in the invisible condition demonstrates that the prime was not processed in a way that could influence the proceeding target. These findings have implications for understanding the impact of subconscious processing on perceptual discrimination.

The presence of priming effects in the visible but not invisible condition could be explained by differences in top-down modulation. Under ordinary circumstances without masking, electrophysiological studies demonstrate that activation latencies in higher-order areas in the prefrontal cortex coincides with or even precedes ventral stream activation in perceptual recognition tasks (Foxe and Simpson, 2002; Michel et al., 2004; Bar et al., 2006). Results like these are often interpreted as reflecting top-down modulation on ventral stream areas (Camprodon et al., 2010). It is known that masking a stimulus interferes with processing in these areas and subsequently diminishes the effects of top-down modulation in such a way that eliminates awareness of the stimulus (Fahrenfort et al., 2007). In the present investigation, the subliminally presented primes did not induce priming effects, which run contrary to models that support feedforward projections (Riesenhuber and Poggio, 2000), and favor instead the importance of interactive feedback and recurrent processing (Rao and Ballard, 1999).

On the other hand, we do not believe that differences in priming effects between the visible vs. invisible conditions could be explained by attention because differences in attention between the two conditions were not possible. The order of conditions was randomly generated and therefore there was no way for the participant to know beforehand if a given condition corresponded to the visible or invisible condition, which would be required to allow them to direct attention differently. This is an important consideration. Selective attention can influence perceptual discrimination (Moore and Zirnsak, 2017). Although neurons in the early stages of visual processing respond robustly and predictably to basic low-level object features (Maunsell and Treue, 2006), they are not solely governed by retinal stimulation. Like neurons in many other brain regions, they too are modulated by attention (Somers et al., 1999).

### Form Experiment

We found no evidence of subliminal priming in the form experiment. Previous experiments using CFS have shown that the subliminal presentation of elongated manipulable tool objects can influence the subsequent classification of visible ones as being either a tool or an animal (Almeida et al., 2008; Sakuraba et al., 2012). However, it was unclear from these studies as to whether or not these effects were driven by the elongated shape of the tools. A more recent CFS study by Hesselmann et al. (2016) demonstrated that an objects’ shape, rather than it semantic category, may have facilitated these effects. The authors failed to replicate these earlier findings in one experiment but they were able to demonstrate in a separate experiment that the suppression of elongated objects from awareness, irrespective of whether they consisted of tools or animals, facilitated the participant’s ability to subsequently classify visible target images as being either elongated or not.

Despite regions in the dorsal visual pathway typically not being associated with object recognition, some research suggests that the unconscious computations mediated by areas in this pathway may influence object categorization (Almeida et al., 2008). This makes sense if one considers that the unconscious processes carried out by the dorsal stream are more concerned with configuring the hand to graspable objects, which tend to be elongated in the real world, and lead to greater fMRI activation in the superior parietal lobule compared to when non-graspable objects are presented (Fang and He, 2005). The findings from the present investigation are consistent with this previous research if one considers that our stimuli across all three experiments were not elongated. In agreement with this earlier work, we did not observe priming for non-elongated stimuli.

However, a different study by Jimenez et al. (2017) demonstrated that the presentation of subliminal square-like and diamond-like stimuli, which are non-elongated shapes, can facilitate their subsequent identification when presented as a visible target. An important difference between their study and ours is that their primes consisted of local elements arranged as either a shape or an illusory contour. In contrast, our study presented primes consisting of a continuous outline of a shape. It is conceivable that the construction of illusory form is likely to utilize different processing mechanisms (Marr, 1982). Alternatively, differences between the studies could have arisen due to differences in the familiarity of the stimuli. Jimenez et al. (2017) used geometrical shapes that are well-known to people while we used geometrical shapes that are less ubiquitous in the environment.

### Orientation Experiment

We also found no evidence of subliminal priming in the orientation experiment. This lack of evidence does not agree with previous demonstrations of masked orientation being discriminable outside of awareness. For instance, Montoro et al. (2014) found that masked global patterns oriented horizontally or vertically primed the orientation of subsequent targets. Participants were told that they would see target lines on the screen and would have to categorize the lines orientation as being either vertical or horizontal. The authors saw reliable differences in reaction time between congruent and incongruent prime-target combinations. Similarly, another study saw that the gross direction of a masked arrow facilitated the subsequent categorization of another arrow (Peremen and Lamy, 2014). However, global compared to local processing is considered to require higher-order processes (Schwarzkopf and Rees, 2011) and thus may not be representative of orientation construction in its most basic sense.

Perhaps the most convincing evidence of orientation processing occurring outside of awareness comes from studies by Soto et al. (2011) and King et al. (2016). The latter repeated the same masking paradigm as the former during magnetoencephalography (MEG). Both studies demonstrated that the orientation of masked stimuli could still be maintained and compared to a later stimulus at levels significantly above chance. The latter also demonstrated that both visible and invisible stimuli activated similar brain areas. However, it should be noted that both studies used higher luminance contrast values than we did in the present investigation. For example, King et al. (2016) used three levels of luminance contrast on all participants, which were 25%, 75% and 100%. Following each trial, participants indicated on a 4-point Likert scale the degree to which the stimulus was visible. From these data, the authors examined behavioral priming and brain activation at each of the different levels. Although the authors demonstrated evidence of subliminal processing both behaviorally and with brain imaging, it should be noted that the contrast values of the trials were at least 25%, providing a stronger signal for the brain to analyze than the stimuli used in the present investigation, which had a mean luminance contrast of 7%. Thus, we applied a more rigorous and conservative procedure to ensure that our stimuli were perceptually invisible. Given the huge difference in luminance contrast between our two studies it could be the case that the Likert scale used by King et al. (2016) lacked the sensitivity to determine which trials were truly subliminal.

### Size Experiment

The size experiment demonstrated that awareness of the prime was important to facilitate the subsequent perceptual size discrimination of the target as evidenced by the presence of priming in the visible but not invisible conditions. This interpretation converges with findings from one of our previous CFS studies (Laycock et al., 2017) and has important implications. FMRI studies reveal that the lateral occipital complex, which is presumably where the current shape stimuli are first processed holistically, consists of two subdivisions—a posterior subdivision that is sensitive to changes in size and an anterior subdivision that is not (e.g., Larsson and Heeger, 2006). Perhaps it is the case that further processing in the more anterior subdivision requires awareness. In addition, other fMRI studies reveal how V1 responds to different sizes of stimuli in a perceived as opposed to a retinotopic manner (Murray et al., 2006; Sperandio et al., 2012; for a review see Sperandio and Chouinard, 2015). These results have often been explained as reflecting top-down modulation from higher-order visual areas—although this has yet to be confirmed (Sperandio and Chouinard, 2015). Based on the lack of priming in the present investigation, it could be the case that awareness and additional ventral stream processing might be necessary for this modulation to occur.

Other masking studies demonstrated how differences in size between the prime and target have little to no impact on how participants respond to the target. For instance, Dehaene et al. (2001) demonstrated that priming effects were similar during a word classification irrespective of when the participants saw masked primes that had similar or different font sizes than the target words. Similarly, Chauncey et al. (2008) revealed how the evoked-response potential (ERP) profiles in early visual areas were the same when participants were presented with different font sizes of the same words during masking. Another study by Eddy and Holcomb (2009) assessed the effect that image size of common objects like cars and couches have on repetition priming during masking. Again, the physical size of the images demonstrated a degree of invariance across the early and later stages of perceptual processing. However, participants in these studies did not distinguish between stimuli on the basis of their size. Rather, these studies sought to determine if priming effects were to persist even with changes in size.

### Limitations

Many of our interpretations rely on drawing inferences from null effects in the invisible conditions. For this reason, it was important for us to include visible conditions, which establishes the presence of effects during awareness, and carry out two statistical approaches (i.e., NHST and Bayesian statistics) to determine the degree of convergence. A greater degree of convergence between two statistical approaches yields greater confidence in the findings, particularly when one (NHST) is fundamentally not designed to verify the absence of effects. With this in mind, we can be more confident about some results than we can for others. Specifically, we can be more confident about reporting the lack of priming in the invisible condition during the Form and Orientation experiments than we can with the Size experiment. Bayesian statistics demonstrated substantial support for the null hypothesis in the invisible condition for both the Form and Orientation experiments but not for the Size experiments. One should always be cautious when making inferences based on null effects, particularly when there is a lack of convergence, such as was the case in the Size experiment. Further investigation is required for confirmation.

### Closing Remarks

This study investigated the extent to which basic low-level object features might be processed outside of awareness for the purposes of perceptual discrimination. It may seem paradoxical that subliminal priming can occur for complex stimuli, as evidenced elsewhere, but not for the simple stimuli used in the present investigation. However, as discussed, these differences may have important theoretical implications and should be examined further. We demonstrate preliminary evidence that subconscious processing does not facilitate subsequent perceptual discrimination task for basic low-level object features.

## Author Contributions

HP, IS, RL and PC designed the study and wrote the manuscript. HP collected the data. HP and PC analyzed the data.

## Conflict of Interest Statement

The authors declare that the research was conducted in the absence of any commercial or financial relationships that could be construed as a potential conflict of interest.
